# Nutrient criteria to achieve New Zealand’s riverine macroinvertebrate targets

**DOI:** 10.7717/peerj.11556

**Published:** 2021-05-31

**Authors:** Adam D. Canning, Michael K. Joy, Russell G. Death

**Affiliations:** 1 Centre for Tropical Water and Aquatic Ecosystem Research, James Cook University, Townsville, Queensland, Australia; 2 School of Government, Victoria University of Wellington, Wellington, New Zealand; 3 School of Agriculture and Environment, Massey University, Palmerston North, New Zealand

**Keywords:** Eutrophication, Nutrient enrichment, Rivers, New Zealand, Macroinvertebrates, Nutrient criteria, Nutrient limits, Nitrogen, Phosphorus

## Abstract

Waterways worldwide are experiencing nutrient enrichment from population growth and intensive agriculture, and New Zealand is part of this global trend. Increasing fertilizer in New Zealand and intensive agriculture have driven substantial water quality declines over recent decades. A recent national directive has set environmental managers a range of riverine ecological targets, including three macroinvertebrate indicators, and requires nutrient criteria be set to support their achievement. To support these national aspirations, we use the minimization-of-mismatch analysis to derive potential nutrient criteria. Given that nutrient and macroinvertebrate monitoring often does not occur at the same sites, we compared nutrient criteria derived at sites where macroinvertebrates and nutrients are monitored concurrently with nutrient criteria derived at all macroinvertebrate monitoring sites and using modelled nutrients. To support all three macroinvertebrate targets, we suggest that suitable nutrient criteria would set median dissolved inorganic nitrogen concentrations at ~0.6 mg/L and median dissolved reactive phosphorus concentrations at ~0.02 mg/L. We recognize that deriving site-specific nutrient criteria requires the balancing of multiple values and consideration of multiple targets, and anticipate that criteria derived here will help and support these environmental goals.

## Introduction

Nutrient enrichment is a key stressor of waterway health across the globe, and is largely driven by population growth, increased fertilizer application and intensive agriculture (Kahiluoto et al., 2014; Nash et al., 2017; McDowell et al., 2020a). Nutrient enrichment can drive excessive algal and microbial growth, which can relieve energy, nutrient and macromolecule growth constraints further up the food web, altering the biological communities (Elser et al., 2000; Benstead et al., 2009; Ferreira et al., 2015; Dodds & Smith, 2016). While autochthonous production by algae can increase the total energy pool available, microbes can condition nutrient-poor detritus, making organic matter more palatable to detritivores, thus enabling another energy source that can also alter biological communities (Elser et al., 2000; Benstead et al., 2009; Ferreira et al., 2015; Dodds & Smith, 2016). In more extreme cases, excessive respiration rates attributed to high autotrophic production, high decomposition rates and altered biological communities can result in hypoxic conditions that drive die-off events, such as mass fish kills, or metabolic toxicity of aquatic organisms (Ferreira et al., 2015; Le Moal et al., 2019; Wurtsbaugh, Paerl & Dodds, 2019; Canning & Death, 2021). Furthermore, experimental nutrient enrichment studies have observed altered ecological communities by permitting the growth of nutrient-limited invertebrates (Kiffney & Richardson, 2001; Cross et al., 2006; Demi et al., 2018).

Macroinvertebrate growth can be limited by ambient nutrient concentrations as they typically have little flexibility (i.e., show strong homeostasis) to adjust body nutrient stoichiometry to accommodate environmental limitations (Elser et al., 2000; Persson et al., 2010; Hessen et al., 2013). Nitrogen limitation can arise from the need to replace nitrogen-rich chitin from moulting exoskeletons and produce protein and nucleic acids (Elser et al., 1996; Frainer et al., 2016). Whilst phosphorus limitation can arise from protein synthesis as this requires P-rich ribosomal RNA (Gillooly et al., 2005; Hessen, Ventura & Elser, 2008; Hessen et al., 2013). Furthermore, the phosphorus content, specific growth rate, DNA and RNA content typically negatively correlate with body size (Elser et al., 2000; Gillooly et al., 2005; Hessen et al., 2013). Nutrients can also limit growth by constraining the availability of macromolecules, such as sterols, essential amino acids and fatty acids provided by nutrient-limited microbes (Mueller-Navarra, 1995; Goedkoop, Demandt & Ahlgren, 2007; Wacker & Martin-Creuzburg, 2012; Guo et al., 2016). Furthermore, experimental nutrient enrichment studies have observed altered ecological communities by permitting the growth of nutrient-limited invertebrates (Kiffney & Richardson, 2001; Cross et al., 2006; Demi et al., 2018). For example, Cross et al. (2005) experimentally enriched a detritus-based pristine stream for two years. Whilst there was no growth effect on the stoneflies, the growth rate of chironomids increased by ~50% and production (by area) increased 183%, which had cascading impacts altering the nutrient stoichiometry of the entire ecosystem (Cross et al., 2003, 2005). Nutrient enrichment can, therefore, result in a dominance of small-bodied, fast-growing invertebrates, such as chironomids and snails (Elser et al., 1996; Frost et al., 2006; Singer & Battin, 2007; Evans-White et al., 2009; Back & King, 2013). Invertebrate assemblages with small-bodied individuals may be less energetically rewarding for fish and may alter fish communities (Schindler & Eby, 1997; Vinson & Baker, 2008; Shearer & Hayes, 2019). Clearly, nutrient enrichment can alter macroinvertebrate communities via multiple mechanisms.

While the mechanisms and impacts of excessive nutrient enrichment of waterways have been well documented (Le Moal et al., 2019; Wurtsbaugh, Paerl & Dodds, 2019; Mallin & Cahoon, 2020), deriving nutrient criteria that are both protective of ecosystems and politically acceptable has proven challenging. Aside from management conflicts due to competing priorities, difficulties typically arise from uncertainty in relationships, discrepancies in criteria between different derivation methods, and a lack of suitable data (Huo et al., 2018; Phillips et al., 2019; Poikane et al., 2021). Often (but not always) setting instream nutrient criteria involves establishing relationships between biological metrics, such as those for macroinvertebrate assemblages, and nutrient concentrations, and then setting nutrient criteria that correspond to desired outcomes (Dodds, 2007; Evans-White, Haggard & Scott, 2013; Huo et al., 2018; Poikane et al., 2019a). While the principle is similar, approaches and metrics for establishing nutrient criteria that correspond to desired macroinvertebrate outcomes have differed considerably between regions. For example, methods include: regression trees and two-dimensional Kolmogorov-Smirnov techniques in Wisconsin, USA (Wang, Robertson & Garrison, 2007; Weigel & Robertson, 2007); Threshold Indicator Taxa Analysis and quantile regression in Central Europe (Kail, Arle & Jähnig, 2012); regressions and regression trees in Ohio, USA (Miltner, 2010); multivariate analysis in Ozark, USA (Justus et al., 2010); cluster analysis in New York State, USA (Smith, Bode & Kleppel, 2007); nonparametric changepoint analysis in Florida, USA (King & Richardson, 2003); regressions in England (Everall et al., 2019); and a compilation of methods based on their weight of evidence in New York State, USA (Smith & Tran, 2010). There is no ‘one size fits all’ approach to deriving nutrient criteria, with methods often reflecting different data availability and the policy framework/ambitions they support. Regression approaches often try to derive nutrient criteria that correspond to desired biological targets, with the desired stringency (and certainty of outcome) dependent on the quantile used for the regression. While regression tree and changepoint methods seek nutrient criteria to prevent reaching ecological tipping points, rather than desired biological targets.

New Zealand (NZ), like many other parts of the world, faces eutrophication of its waterways, primarily from the intensification of agriculture. Fertilizer use in NZ has increased by 627% between 1990 and 2015, with 70% of rivers, by length, experiencing nutrient enrichment above natural levels, and many sites in declining condition (Ministry for the Environment & Statistics New Zealand, 2020). Despite the declining water quality, the implementation and enforcement of suitable nutrient criteria have been the subject of intensive debate, with little resolution in recent national freshwater policy reforms (Joy & Canning, 2020).

The development of suitable nutrient criteria for New Zealand’s rivers and streams has, in part, been constrained by a lack of suitable data (Death et al., 2018; Canning, 2020). The monitoring of New Zealand’s waterways is non-random, often reflecting points of interest and the objectives of environmental managers. As a result, many nutrient monitoring sites do not have biological monitoring and vice-versa, constraining the ability to form relationships between nutrients and biological responses. This limited dataset comprising only sites where both nutrients and biological monitoring occurs simultaneously may fail to encapsulate the range of possible responses, particularly if different river geomorphologies, land uses, or nutrient concentrations are under-represented (Canning, 2020). One way to circumvent the mismatch of data could be to use data predicted from national water quality models that cover all river reaches (Whitehead, 2018). While modelled data may fail to encapsulate localised nuances, advantages may include the smoothing of noisy data and full representation across the entire river network (Özkundakci et al., 2018). In-situ nutrient monitoring data is notoriously prone to high variability, with grab samples often influenced by hydrological patterns, geology, temporal variability in land uses/practices (e.g., time of fertiliser application), variability in biological uptake from diurnal processes and predator-prey cycling, and variation in other physicochemical conditions (e.g., dissolved oxygen and temperature) (e.g., Jordan, Correll & Weller, 1997; Glibert et al., 2008; Aguilera, Marcé & Sabater, 2012). Examining the differences in nutrient criteria derived from using measured data and from modelled data may build confidence in derived criteria if there is strong convergence or help elucidate whether further data collection is required.

In moves seeking to improve the health of the nation’s freshwaters, the New Zealand Government developed the *National Policy Statement for Freshwater Management (NPS-FM 2020)*. This new national policy prescribes three riverine macroinvertebrate indicators (termed ‘attributes’) of ecological health: (1) the Macroinvertebrate Community Index (MCI; Stark & Maxted, 2007); (2) the quantitative variant of the MCI (QMCI; Stark & Maxted, 2007); and (3) the average score per metric (ASPM) (Collier, 2008). The MCI and QMCI indicate the overall community’s sensitivity to organic enrichment from weighted averages of species tolerance scores from presence-absence invertebrate surveys (MCI) or relative abundance surveys (QMCI). The ASPM is an overall indicator of community health and is the normalised average of the richness of Ephemeroptera, Plecoptera and Trichoptera taxa excluding Hydroptilidae (EPT), % EPT and the macroinvertebrate community index (MCI) (Collier, 2008). The NPS-FM (2020) requires local authorities to improve the attributes for each river to at least the ‘national bottom line’ or better. The national bottom line targets are described by the NPS-FM (2020) as being broadly indicative of a moderate level of ecological integrity and organic pollution, with a mix of taxa sensitive and insensitive to organic pollution/nutrient enrichment. Local authorities must then also set nutrient criteria for dissolved inorganic nitrogen (DIN) and dissolved reactive phosphorus (DRP) at concentrations appropriate for achieving desired outcomes. Ideally, nutrient criteria would not be more stringent than required to support the desired ecological outcome (i.e., biology pass, yet nutrients fail prescribed criteria), or too weak to support the desired ecological outcome (i.e., biology fail, yet nutrients pass prescribed criteria).

The minimisation of mismatch between nutrients and biology (‘minimisation-of-mismatch’) approach, as described by the European Union’s ‘Best practice for establishing nutrient concentrations to support good ecological status’ guidelines, provides an objective and robust method for deriving nutrient criteria (Phillips et al., 2018, 2019). The minimisation-of-mismatch approach seeks to identify nutrient criteria that are most likely to pass or fail when the ecological indicators also pass or fail respectively, is little affected by weak, nonlinear biology-nutrient regressions or data distribution. Minimisation-of-mismatch avoids the adoption of arbitrary percentiles that quantile regression approaches require; while changepoint analysis approaches are unable to benchmark nutrient criteria against pre-defined biological criteria–whereas minimisation-of-mismatch can (Phillips et al., 2018, 2019). Poikane et al. (2019b) applied the method to derive nutrient criteria to support healthy European lakes and found the approach yielded similar nutrient criteria to the other methods examined. Broad agreement with other methods, tolerance of data structures, and the avoidance of arbitrary decision-making, would make the method attractive to decision-makers faced with politically contentious issues.

Here we aim to use the minimisation-of-mismatch approach to derive nutrient criteria for dissolved inorganic nitrogen (DIN) and dissolved reactive phosphorus (DRP) that support the achievement of the national bottom line targets for the three riverine macroinvertebrate attributes, as stipulated by New Zealand’s NPS-FM 2020. Given that New Zealand’s monitoring data is largely non-random (with some localised examples of randomised surveys), with nutrients measured at all biological monitoring sites, we also aim to compare the nutrient criteria derived when modelled nutrient data is used instead of measured data.

## Materials & methods

### Macroinvertebrate and nutrient data

**Figure 1:**
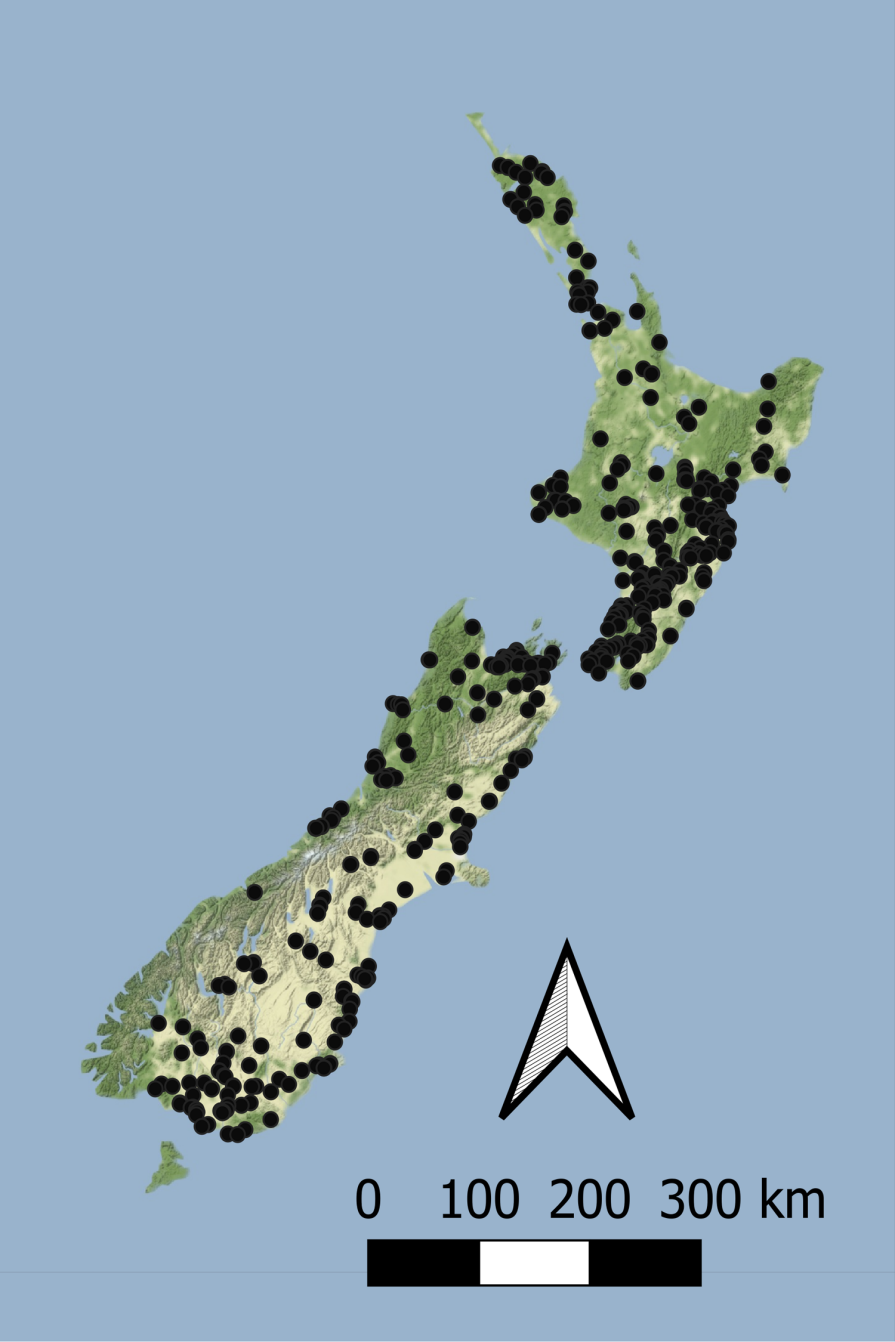
The location of sites with concurrent macroinvertebrate and nutrient monitoring across New Zealand. Sites are surveyed as part of New Zealand’s state of environment monitoring between 2013 & 2017 (Ministry for the Environment & Statistics New Zealand, 2019). Macroinvertebrates are surveyed annually and nutrients are sampled monthly.

Macroinvertebrate data used in this study was sourced from New Zealand’s regional environmental monitoring network of rivers and streams (Ministry for the Environment & Statistics New Zealand, 2019). Benthic macroinvertebrates were surveyed annually for five years during summer by regional authorities between 2012 and 2016, and the MCI, QMCI and ASPM scores calculated using a consistent taxonomic resolution by Clapcott et al. (2017). Macroinvertebrates were typically sampled in riffles using either kick nets or Surber samplers, with five to seven replicates, stored in either ethanol or formalin, and identified using common keys (e.g., Winterbourn, Gregson & Dolphin, 1989; Moore, 1998). Surber sampling aimed to capture all invertebrates within a 0.1 m^2^ area to a depth of ~10 cm, whereas kick net sampling typically involved shuffling gravel for ~1–2 min until several hundred invertebrates are collected. While kick nets and Surber samplers may result in different invertebrate assemblages being collected, investigations by Stark (1993) suggest that differences in MCI and taxa richness are rarely significant. Surveys were collected from 1851 sites nationwide (Fig. 1).

DIN and DRP monitoring data was also sourced from New Zealand’s regional environmental monitoring network of rivers and streams (Ministry for the Environment & Statistics New Zealand, 2019). Between 2012 and 2016, grab samples testing DIN and DRP were collected monthly at 856 and 878 sites respectively, and summarised over the entire five-year period as a median for each site.

All modelled nutrient data was sourced from Whitehead (2018), and is also used in national environmental reporting (Ministry for the Environment & Statistics New Zealand, 2019). Modelled nutrient data includes predictions of DIN (calculated as NO_3_-N + NH_4_-N) and DRP for every river reach across New Zealand’s riverine network (*N* = 593,373 reaches).

Two datasets were compiled for this analysis: (1) measured MCI, QMCI and ASPM scores at sites with DIN and DRP concentrations measured concurrently (*N*_MCI_ = 450, *N*_QMCI_ = 294, & *N*_ASPM_ = 389; Table 1); and (2) measured MCI, QMCI and ASPM scores at all invertebrate survey sites (regardless of whether nutrients were measured or not) with modelled DIN and DRP concentrations (*N*_MCI_ = 1,729, *N*_QMCI_ = 1,450, & *N*_ASPM_ = 1,729; Table 1).

**Table 1:** Summary statistics of raw invertebrate and nutrient data. The minimum, median, mean, maximum, 25th and 75th percentiles of MCI, QMCI, ASPM, measured DIN and DRP (ug/L), and modelled DIN and DRP across New Zealand’s state of environment monitoring network data used in this analysis.

Dataset	Metric	Statistic					
		Min	25th percentile	Median	Mean	75th percentile	Max
Measured	MCI	54.8	91.0	103.5	103.2	116.0	148.0
	QMCI	2.0	4.1	5.0	5.1	6.0	7.9
	ASPM	0.11	0.33	0.44	0.42	0.52	0.78
	DIN	1.0	51.5	241.0	567.3	670.0	1,0578.8
	DRP	0.3	5.0	9.5	16.1	16.0	250.0
Modelled	MCI	34.8	88.4	104.1	103.0	118.0	161.3
	QMCI	2.0	4.3	5.6	5.7	6.6	106.2
	ASPM	0.07	0.28	0.43	0.41	0.54	0.83
	DIN	10.9	79.2	224.2	397.4	567.3	5,215.6
	DRP	1.1	7.7	12.6	15.3	20.2	109.7

To evaluate the efficacy of the nutrient models, using R 3.5.3 (R Development Core Team, 2019) linear regression analysis was used to examine the ability of the modelled nutrients to reflect the concentrations measured at sites in dataset (1). As a pre-cursor to the minimization-of-mismatch analysis, regression analysis was carried out between nutrient concentrations and the ecosystem health metrics for both datasets to ascertain the direction of change with nutrient enrichment, as recommended by Phillips et al. (2018).

### Minimization-of-mismatch analysis

Using both datasets, minimization-of-mismatch analysis was used to estimate the DIN and DRP concentrations that corresponded to the national bottom line targets, as per the NPS-FM 2020, for each metric (MCI = 90, QMCI = 4.5, & ASPM = 0.3). The national bottom line targets used in the NPS-FM were established based on the advice on the advice of the Scientific and Technical Advisory Group informing the policy development framework, as informed by a review of macroinvertebrate indicators (Clapcott et al., 2017; Essential Freshwater Science & Technical Advisory Group, 2019). Minimization-of-mismatch analysis estimates the nutrient concentration target that maximizes the probability of a site passing both the ecological metric target and the nutrient concentration target, while seeking to minimize the passing of the ecological target and failing on the nutrient target (vice-versa)–i.e., the mismatch in passing and failing grades is minimized (Phillips et al., 2018). The approach involves three steps: (1) plotting the percentage of water bodies that have a passing score for an ecosystem health metric but a failing nutrient status for different potential nutrient criteria values; (2) then plotting a similar, but inverse, plot with the percentage of water bodies where the ecosystem health metric fails but the nutrient criteria would pass; and then (3) identifying the intersect between the two plots, this indicates the nutrient concentration that minimizes the mismatch of passing and failing grades. For each relationship, this was repeated 1500 times with a random sub-sample using 75% of the total data (with replacement), with the median (mean, range and quantiles also calculated) representing the final nutrient criteria (Phillips et al., 2018, 2019; Poikane et al., 2019b).

## Results

**Figure 2:**
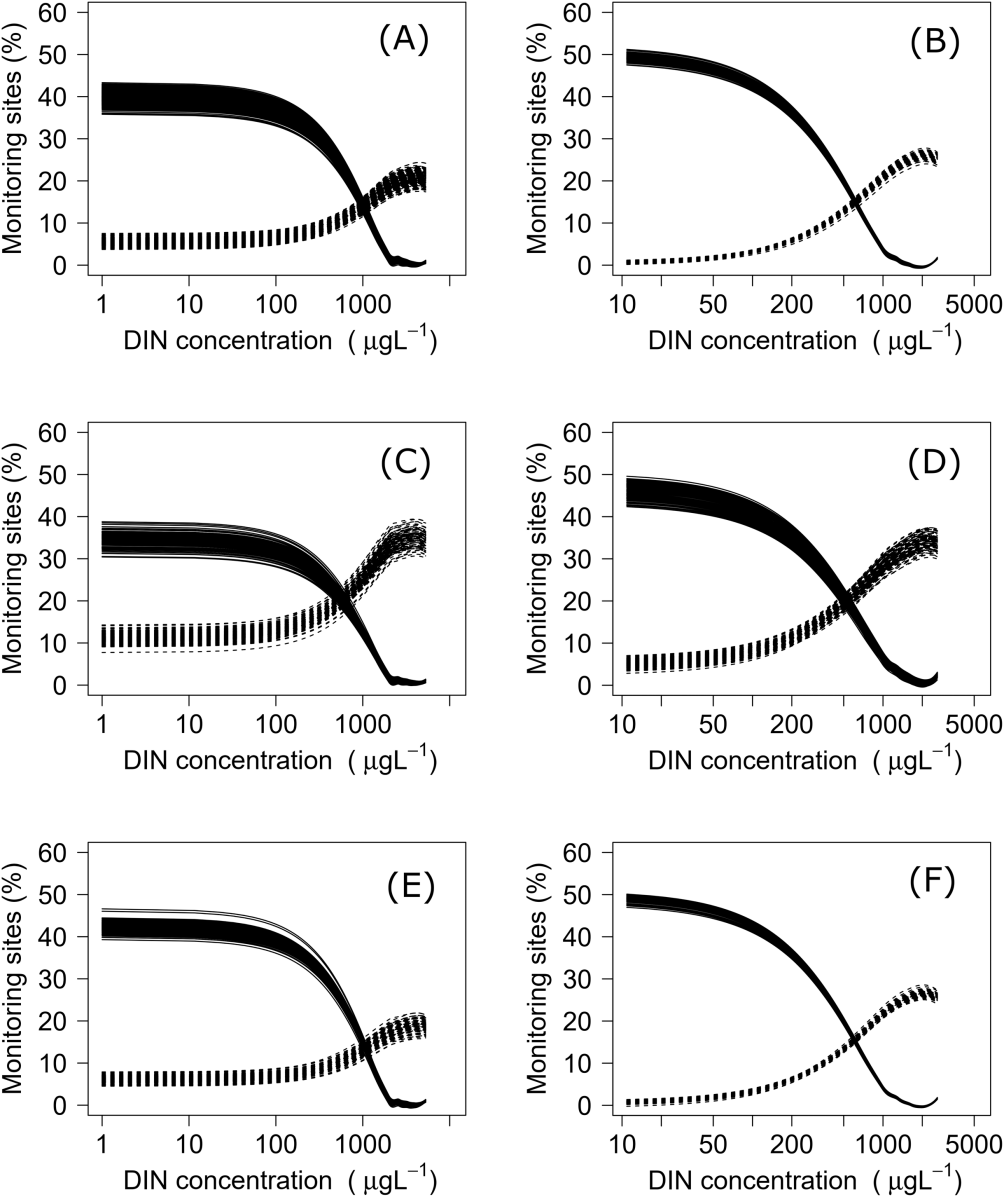
Minimisation-of-mismatch analysis to derive DIN criteria that support New Zealand’s national macroinvertebrate targets. The proportion of water bodies that pass for ecosystem health but fail for nutrients (full line) and the proportion failing ecosystem health and passing DIN (dashed). A & B represent MCI targets, C & D represent QMCI targets, and E & F represent ASPM targets, using measured and modelled nutrients respectively.

**Figure 3:**
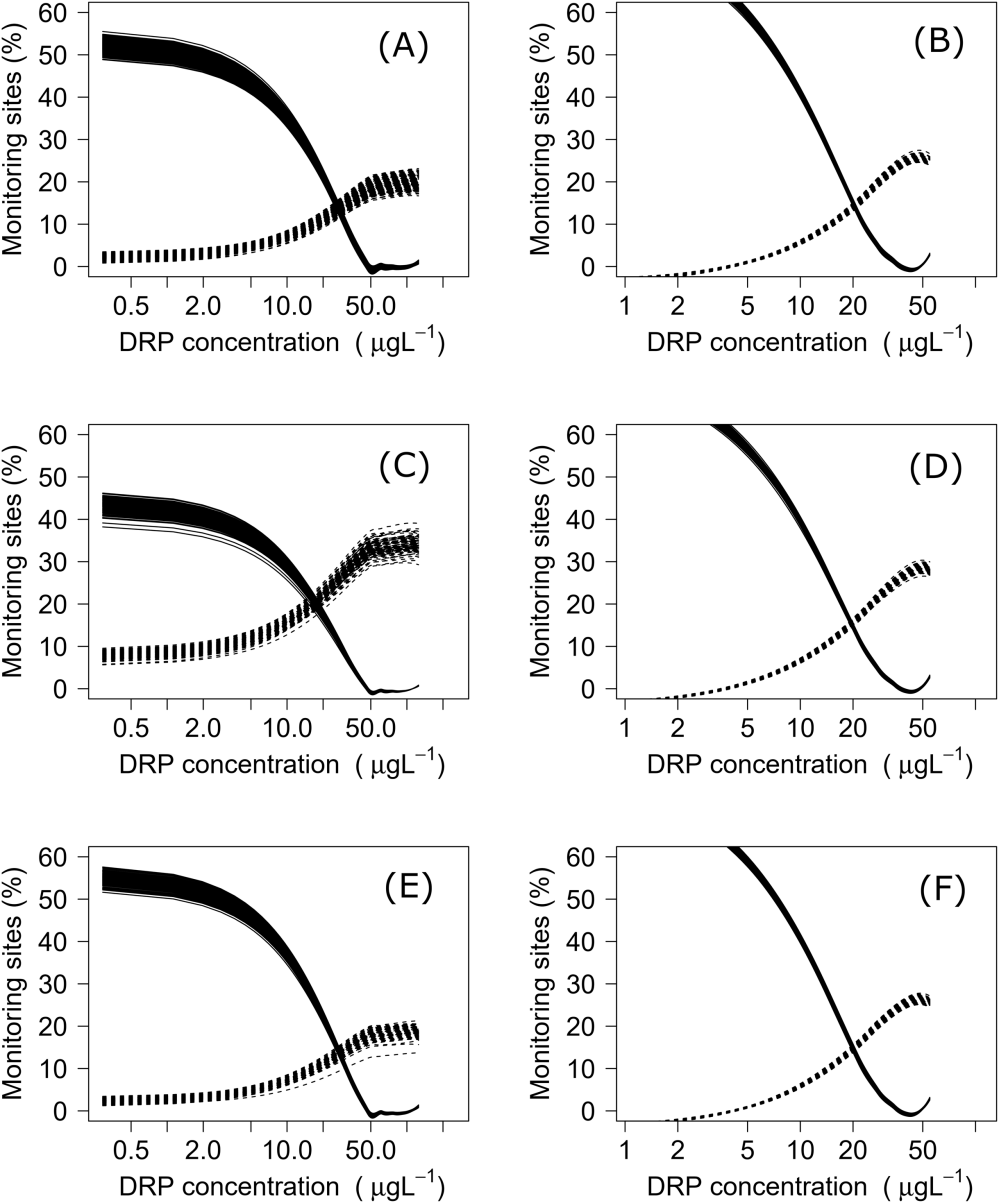
Minimisation-of-mismatch analysis to derive DRP criteria that support New Zealand’s national macroinvertebrate targets. The proportion of water bodies that pass for ecosystem health but fail for nutrients (full line) and the proportion failing ecosystem health and passing DRP (dashed). A & B represent MCI targets, C & D represent QMCI targets, and E & F represent ASPM targets, using measured and modelled nutrients respectively.

Negative relationships were observed between all nutrient and ecosystem health metrics regressions, regardless of dataset (Figs. S1 & S2; Table S1). Across the sites with measured nutrients, mismatch minimization analysis suggests, using the median (range), the best DIN criteria to achieve the national bottom lines for MCI, QMCI and ASPM were 1.07 (0.93–1.21) mg/L, 0.62 (0.46–0.77) mg/L and 1.12 (1.00–1.29) mg/L respectively, while the DRP criteria were 0.028 (0.025–0.030) mg/L, 0.018 (0.015–0.020) mg/L and 0.028 (0.026–0.032) mg/L respectively (Table 2; Fig. 2). While the nutrient criteria derived using all sites and using modelled nutrients suggests, using the median, the best DIN criteria to achieve the national bottom lines for MCI, QMCI and ASPM were 0.64 (0.60–0.68) mg/L, 0.59 (0.57–0.63) mg/L and 0.63 (0.59–0.65) mg/L respectively, while the DRP criteria were 0.021 (0.020–0.021) mg/L, 0.020 (0.195–0.021) mg/L and 0.021 (0.020–0.021) mg/L respectively (Table 2; Fig. 3). Modelled nutrients were highly correlated to measured nutrients at sites with concurrent macroinvertebrate monitoring (DIN: R^2^ = 0.90, F_1,444_ = 4,146, *P* < 0.001; DRP: R^2^ = 0.89, F_1,445_ = 3,589, *P* < 0.001).

**Table 2:** Nutrient criteria to support New Zealand’s national bottom line riverine macroinvertebrate targets. Statistics summarizing the DIN and DRP criteria (mg/L) produced using the minimization-of-mismatch method to support New Zealand’s three macroinvertebrate national bottom lines set out in the NPS-FM 2020.

Nutrient dataset	Metric	Nutrient	Statistic					
			Min	Lower quartile	Median	Mean	Upper quartile	Max
Measured	MCI	DIN	0.93	1.04	1.07	1.07	1.10	1.21
		DRP	0.025	0.027	0.028	0.028	0.028	0.030
	QMCI	DIN	0.46	0.57	0.63	0.62	0.67	0.77
		DRP	0.015	0.017	0.018	0.018	0.019	0.020
	ASPM	DIN	1.01	1.09	1.12	1.13	1.16	1.29
		DRP	0.026	0.028	0.028	0.028	0.029	0.032
Modelled	MCI	DIN	0.60	0.63	0.64	0.64	0.65	0.68
		DRP	0.020	0.021	0.021	0.021	0.021	0.021
	QMCI	DIN	0.01	0.02	0.02	0.02	0.02	0.02
		DRP	0.019	0.020	0.020	0.020	0.020	0.021
	ASPM	DIN	0.59	0.62	0.63	0.63	0.64	0.65
		DRP	0.020	0.020	0.021	0.021	0.021	0.021

## Discussion

When using measured nutrient data, the nutrient criteria derived to support achieving the national bottom line targets for QMCI were more stringent than those for MCI and ASPM. Although the MCI and QMCI are often correlated and similar criteria would be anticipated (Wright-Stow & Winterbourn, 2003), the QMCI may be more sensitive to change as it accounts for changes in species abundance, whereas MCI and ASPM scores only change when a species becomes present or absent entirely. Interestingly, when the larger macroinvertebrate dataset was used with modelled nutrients, the nutrient criteria across all three metrics converged very closely to give a DIN criteria at ~0.6 mg/L and DRP criteria at ~0.02 mg/L–similar to those derived for the QMCI using measured nutrients. The criteria derived using the measured nutrient dataset were, however, more variable than those derived using modelled nutrients. By using modelled nutrients, a larger invertebrate metric dataset was permissible, potentially allowing for greater refinement of nutrient criteria and mitigating against the uncertainty arising from the presence-absence based indicators.

**Figure 4:**
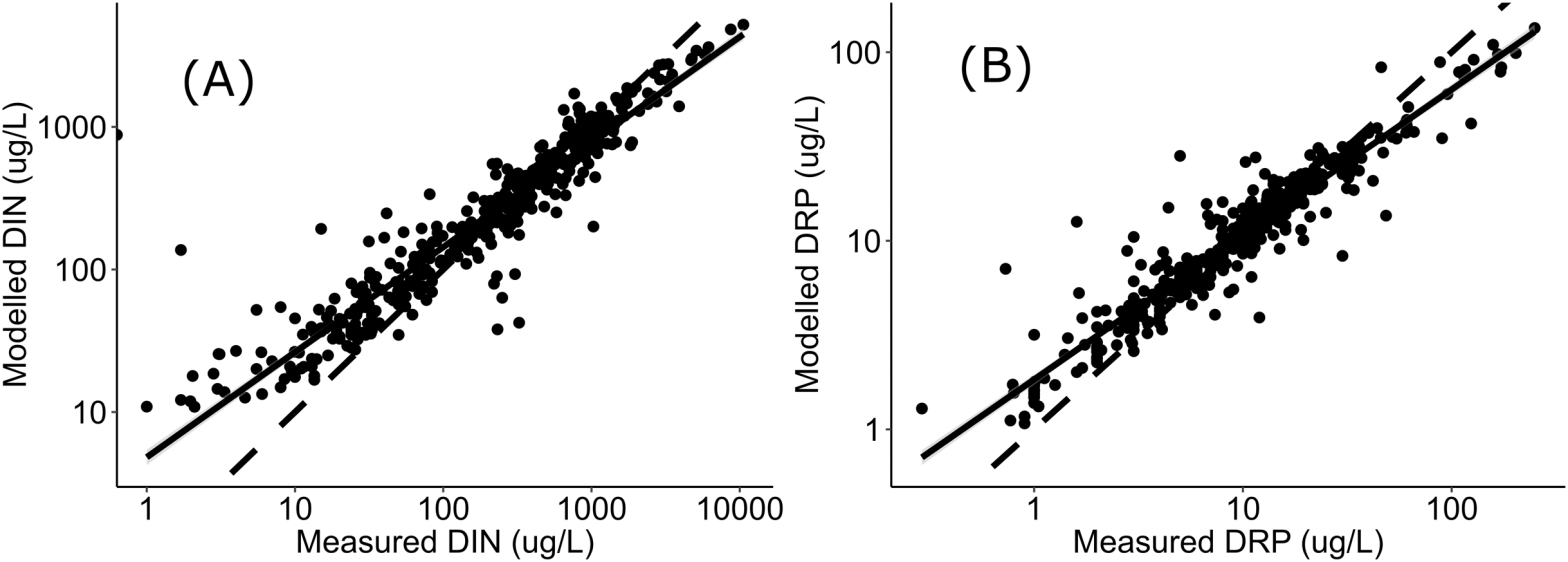
Regressions between measured and modelled nutrient concentrations. Regressions (log-log transformed) between (A) modelled DIN and measured DIN, and (B) modelled DRP and measured DRP at all sites in Data S1 and Fig. 1. Data sourced from New Zealand’s state of environment monitoring between 2013 & 2017 (Ministry for the Environment, Statistics New Zealand, Environment M for the Zealand SN, 2019).

The divergence in criteria between those produced with the measured versus modelled nutrient concentrations, may also arise from uncertainty in estimating high nutrient concentrations. In-situ nutrient concentrations are highly variable, particularly influenced by season and rainfall, with the range of variability typically increasing with increased nutrient concentrations (e.g., Jordan, Correll & Weller, 1997; Glibert et al., 2008; Aguilera, Marcé & Sabater, 2012). The modelled concentrations were well correlated to measured concentrations, as observed in Fig. 4, the modelled concentrations systematically under-estimate high concentrations and over-estimate low concentration. This may be attributed to having fewer residuals at the extremities to anchor the model, leading to greater uncertainty when predicting high concentrations. Incorporating more data from new sites with very high nutrient concentrations may help increase the confidence of derived nutrient criteria; however, this was not available at the time of analysis, and finding numerous highly enriched sites may not be possible if they do not exist. The level of uncertainty accepted for environmental management will inevitably depend on the values guiding the management and policy framework they sit within. If a precautionary approach to environmental management of national bottom lines is desired, then this analysis suggests the DIN and DRP criteria would need to be ~0.6 mg/L and ~0.02 mg/L respectively, or better, particularly given that both measured and modelled nutrient datasets both yielded similarly stringent criteria for supporting the QMCI targets. The nitrogen criteria derived here also fit within the range recommended by Camargo & Alonso (2006), who conducted a global review of inorganic nitrogen pollution in rivers and suggested levels should be less than 0.5-1 mg/L to prevent eutrophication and protect against toxicity.

In addition to the differences arising from sample size and whether measured or modelled nutrients are used, variability in nutrient criteria can also arise from unaccounted external environmental influences and normative decisions made in the derivation process. While nutrient enrichment is highly influential in driving New Zealand’s macroinvertebrate assemblages, other factors such as sedimentation, riparian habitat, pesticides, water hardness, pH, temperature, flow and substrate composition are also influential (Wagenhoff, Townsend & Matthaei, 2012; Burdon, McIntosh & Harding, 2013; Death et al., 2015; Matthaei & Piggott, 2019; Salis et al., 2019). In consequence, these factors may be limiting or altering the restoration of macroinvertebrate communities, and failure to address other stressors may result in the desired ecological outcomes not being realized. While uncertainty can also arise from nutrient grab samples, as nutrients can fluctuate diurnally, and seasonally with rainfall patterns and agricultural practices (Withers & Jarvie, 2008; Causse et al., 2015). Therefore, an assessment period too short may be highly influenced by temporal variability, while variability in very long assessment periods may arise from an improving or declining trend – consequently, deciding the period of assessment to ascertain the state and assess against criteria presents a balance. In this study, grade assessment was made against data summarised at a site over a five-year period, in line with New Zealand’s NPS-FM (2020) and State of Environment reporting (Ministry for the Environment & Statistics New Zealand, 2019). It is also encouraging that the ranges, particularly the interquartile ranges, of nutrient criteria produced for each metric were generally low, suggesting the criteria would be broadly applicable.

Other published nutrient criteria derived for New Zealand rivers include those from the Australia and New Zealand Guidelines (ANZG), those derived by models to support periphyton objectives (Biggs, 2000; Biggs & Kilroy, 2000; Snelder, Moore & Kilroy, 2019), and those derived for macroinvertebrates within the Manawatu-Whanganui region (Wagenhoff et al., 2017a, 2017b; Canning & Death, 2021). The nutrient criteria derived here are considerably more lenient than the ANZG criteria. The ANZG criteria for NZ rivers are derived from the arbitrary percentile of nutrient concentrations predicted to occur in reference conditions for different river typologies. The ANZG criteria are not intended to support a desired ecological state but are to trigger the indication of nutrient concentrations significantly greater than natural state. The nutrient criteria required to achieve the national bottom line targets for periphyton were generally more stringent than those derived here to support the macroinvertebrate metrics, though they vary substantially depending on river geology and hydrology. While explorations using data from the Manawatu-Whanganui region suggest the impact of nutrients on various macroinvertebrate community metrics, macroinvertebrate assemblage turnover, and metrics of food web function, ceased at nitrogen concentrations in excess of 0.5 mg/L – similar, but slightly more stringent than those derived here (Wagenhoff et al., 2017a, 2017b; Canning & Death, 2021). Adopting any of these alternative nutrient criteria would likely provide sufficient nutrient protection to support achieving any of the three macroinvertebrate national bottom line target states, though the relative stringency of alternative nutrient criteria would vary with hydrology, geology and values being managed.

While other jurisdictions have developed nutrient criteria for separate eco-regions (e.g., Zhou & Zheng, 2008; Chambers et al., 2012; USEPA, 2019), we consider that New Zealand likely falls within its own eco-region and did not consider it necessary to develop eco-regionalized nutrient criteria. Given New Zealand’s small size, mountainous geology, short-run rivers and highly variable flood frequency, previous studies have shown that New Zealand rivers share a common core assemblage of macroinvertebrates that are ecologically flexible and have poorly synchronised life histories (Winterbourn, Rounick & Cowie, 1981; Quinn & Hickey, 1990; Thompson & Townsend, 2000). Previous explorations using machine learning have also shown that nutrient concentrations and land use are, by considerable margins, the most influential variables predicting the three metrics, despite an array of other potential predictors covering hydrology, geology and climate (Clapcott et al., 2012, 2017; Canning, 2020). A New Zealand freshwater macroinvertebrate eco-region is also considerably smaller than those in other regions. For example, the eco-regions for USA rivers are, on average, 13 times larger than New Zealand (USEPA, 2019), while those described in China are six times larger (Zhou & Zheng, 2008). Splicing New Zealand into smaller eco-regions when deriving nutrient criteria would mean substantially fewer sites. These sites may fail to encapsulate the range of possible responses, particularly if different river geomorphologies, land uses, or nutrient concentrations are under-represented and yield more uncertain criteria than that from a larger, nationally derived dataset (Canning, 2020). Finally, the mismatch rates were low, ranging between 10–15% (Figs. 2 & 3), and ranges of derived criteria generally narrow, suggesting that the nutrient criteria produced correspond well to the desired biological outcomes for most rivers.

While the nutrient criteria derived here will be useful for supporting the achievement of national macroinvertebrate targets, New Zealand’s National Policy Statement for Freshwater Management (2020) also requires regional authorities to set nutrient criteria to support achievement of other ecological and social targets, which can vary between rivers. Setting nutrient criteria is a multi-disciplinary challenge, involving the political judgement of the implications arising from multiple potential criteria designed to support different ecological, social, cultural, and economic values and objectives. For example, more stringent criteria may be required to achieve other ecological objectives, such as those for algal biomass and dissolved oxygen. Social values affected by nutrient criteria may include recreation (e.g., sports fishing, swimming and boating), identity (i.e., how society views, and connects with, the environment as part of their identity), or human health. For example, a recent meta-analysis observed a statistically significant positive association between nitrate exposure and risk of colorectal cancer (Temkin et al., 2019). Cultural values, such as traditional practices (e.g., indigenous food collection) and New Zealand’s ‘*Te Mana o Te Wai’*, may also affect nutrient criteria. *Te Mana o Te Wai* is a Māori (New Zealand Indigenous) concept that is now embedded within, and mandated by, New Zealand’s NPS-FM (2020), refers to the continued protection and restoration of water and sets the priority by which water use must occur (Te Aho, 2018). *Te Mana o Te Wai* mandates that the health and wellbeing of waterways must be prioritized first, followed by the needs of people (e.g., drinking water), and then the social, cultural and economic needs (Te Aho, 2018; Essential Freshwater Kahui Wai Maori Advisory Group, 2019). While economic values affected by nutrient criteria may include the extent to which productive land can be farmed intensively or the extent to which New Zealand’s ‘clean and green’ branding is improved, maintained, or eroded (Tait et al., 2013; Foote, Joy & Death, 2015; McDowell et al., 2020b). The integration of these values influenced by nutrient criteria, the levels of precaution they require, and the implications within the legislative framework, are beyond the scope of this paper but are clearly important considerations.

## Conclusions

New Zealand’s waterways have been experiencing considerable and widespread nutrient enrichment. We use the minimization-of-mismatch analysis to derive nutrient criteria that could support achievement of national aspirations for three macroinvertebrate indicators of riverine health. This analysis suggests that median DIN concentrations of ~0.6 mg/L and median DRP concentrations of ~0.02 mg/L would be suitable for supporting macroinvertebrate targets.

## Supplemental Information

10.7717/peerj.11556/supp-1 Supplemental Information 1 Regressions between MCI, QMCI and ASPM versus measured DIN and measured DRP. Regressions between MCI, QMCI and ASPM (means from annual surveys between 2012-2016) versus measured DIN and measured DRP (medians from monthly samples between 2012-2016) at all riverine state of environment monitoring sites across New Zealand where both benthic invertebrates and nutrients were sampled concurrently.

10.7717/peerj.11556/supp-2 Supplemental Information 2 Regressions between MCI, QMCI and ASPM versus modelled DIN and modelled DRP. Regressions between MCI, QMCI and ASPM (means from annual surveys between 2012-2016) versus modelled DIN and modelled DRP (Whitehead, 2018) at all riverine state of environment monitoring sites across New Zealand where benthic invertebrates were surveyed. and nutrients were sampled concurrently.

10.7717/peerj.11556/supp-3 Supplemental Information 3 Regression statistics for correlations between ecosystem health metrics and nutrient concentrations. Regression (y~ln(x)) statistics for correlations between ecosystem health metrics and nutrient concentrations, as shown in Figs. S1 & S2. D.F. = degrees of freedom.

10.7717/peerj.11556/supp-4 Supplemental Information 4 Raw nutrient and macroinvertebrate data.

10.7717/peerj.11556/supp-5 Supplemental Information 5 Minimisation-of-mismatch derived criteria for various macroinvertebrate target states. While beyond the scope of this study, nutrient criteria derived using the minimisation-of-mismatch method for alternative macroinvertebrate target states (indicated by the MCI, QMCI and ASPM) across New Zealand rivers

## Additional Information and Declarations

### Data Availability

The following information was supplied regarding data availability: Data are available in the Supplemental Files.
