# Supplementary figures and images for: Nutrient criteria to achieve New Zealand’s riverine macroinvertebrate targets
Source: PeerJ. 2021 May 31;9:e11556. doi: 10.7717/peerj.11556 (PMC8174153; doi:10.7717/peerj.11556)

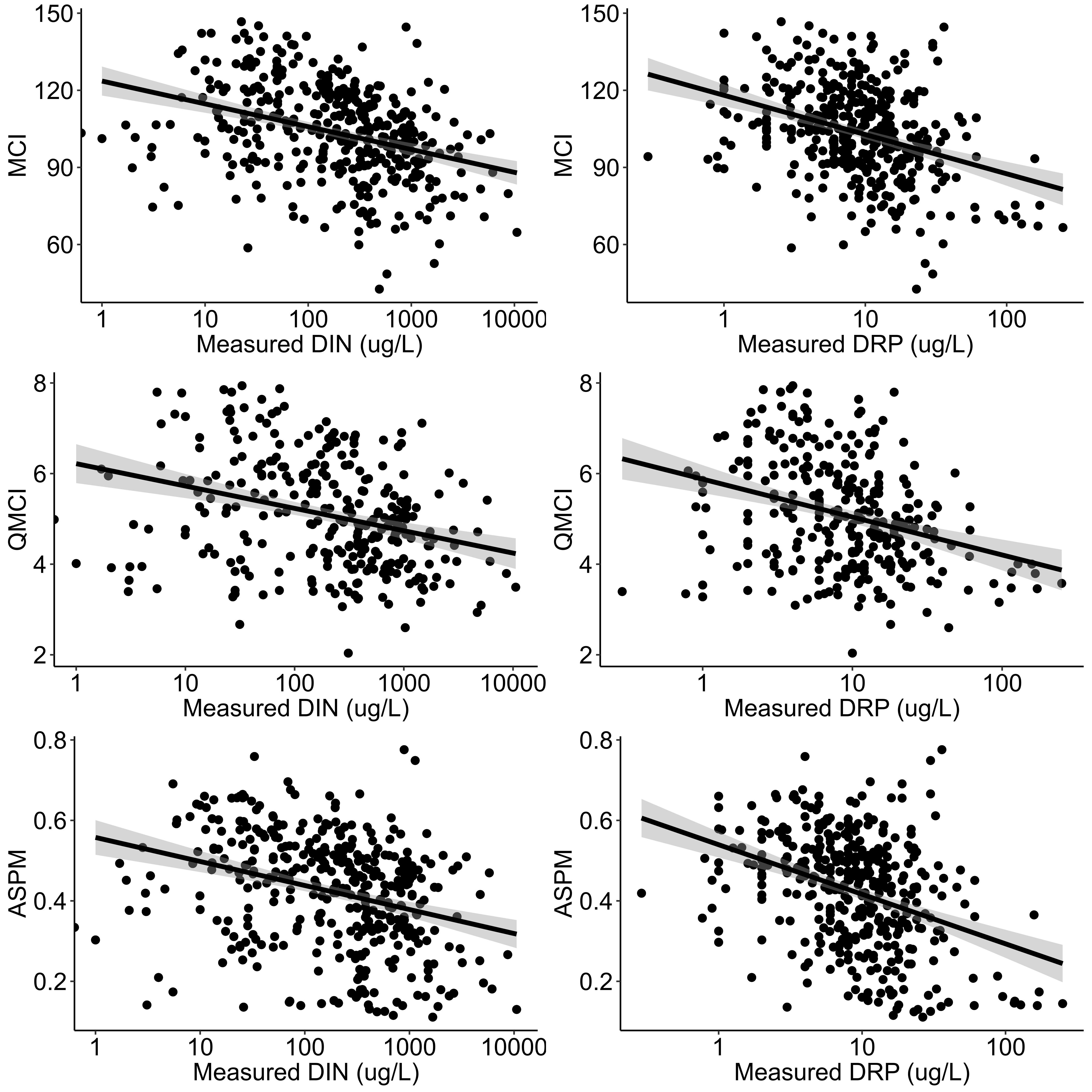

Supplement: Supplemental Information 1 — Regressions between MCI, QMCI and ASPM (means from annual surveys between 2012-2016) versus measured DIN and measured DRP (medians from monthly samples between 2012-2016) at all riverine state of environment monitoring sites across New Zealand where both benthic invertebrates and nutrients were sampled concurrently. [file peerj-09-11556-s001.png]

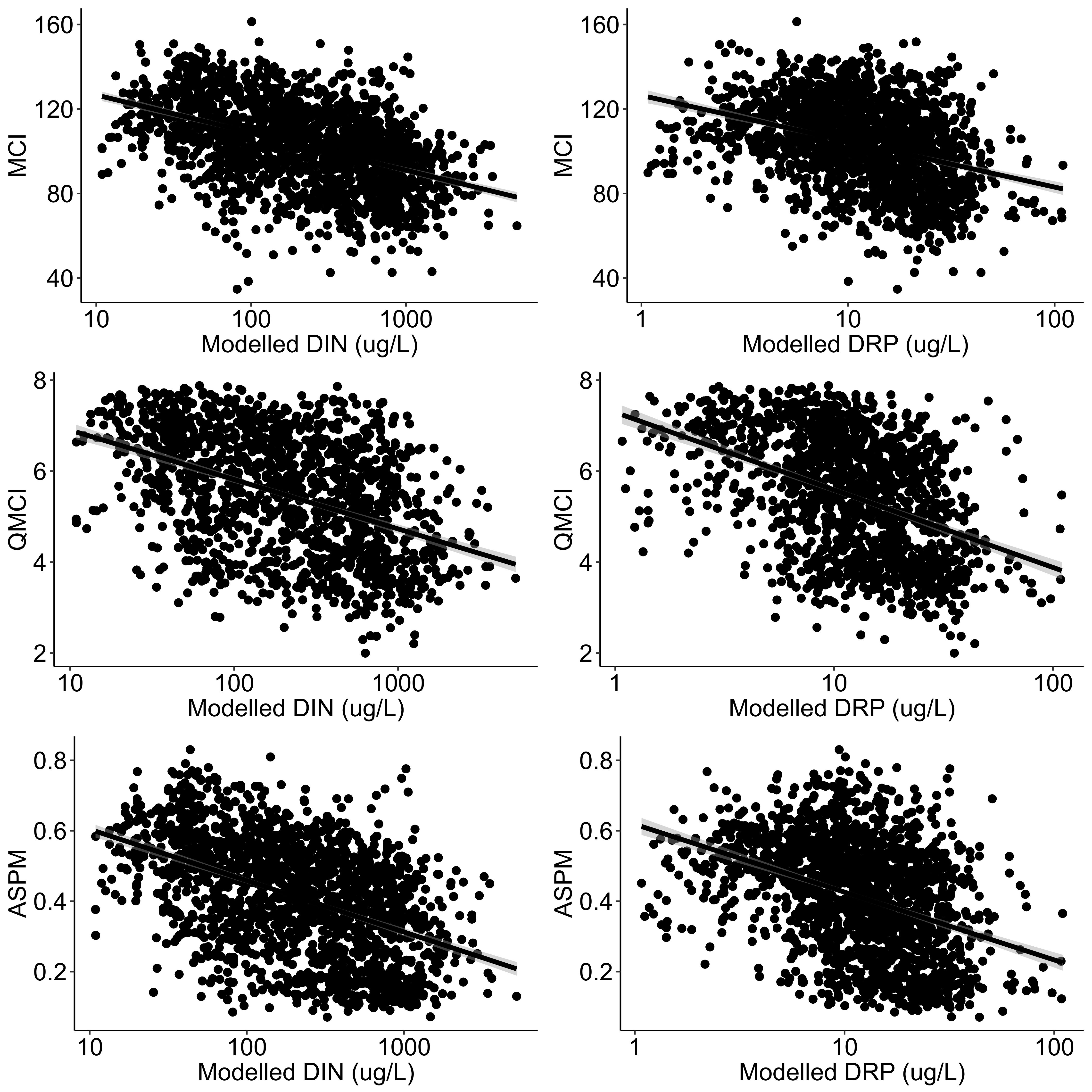

Supplement: Supplemental Information 2 — Regressions between MCI, QMCI and ASPM (means from annual surveys between 2012-2016) versus modelled DIN and modelled DRP (Whitehead, 2018) at all riverine state of environment monitoring sites across New Zealand where benthic invertebrates were surveyed. and nutrients were sampled concurrently. [file peerj-09-11556-s002.png]
