# Supplementary material for: Nutrient criteria to achieve New Zealand’s riverine macroinvertebrate targets
Source: PeerJ. 2021 May 31;9:e11556. doi: 10.7717/peerj.11556 (PMC8174153; doi:10.7717/peerj.11556)
Supplement: Supplemental Information 3 — Regression (y~ln(x)) statistics for correlations between ecosystem health metrics and nutrient concentrations, as shown in Figs. S1 & S2. D.F. = degrees of freedom. [file peerj-09-11556-s003.docx]

| Nutrient estimation | Nutrient | Metric | R^2^ | F-stat | D.F. | P-value |
| --- | --- | --- | --- | --- | --- | --- |
| Measured | DIN | MCI | 0.11 | 61.93 | 1 & 447 | 2.7E^-14^ |
|  |  | QMCI | 0.08 | 23.28 | 1 & 291 | 2.3E^-6^ |
|  |  | ASPM | 0.08 | 31.21 | 1 & 386 | 4.4E^-8^ |
|  | DRP | MCI | 0.10 | 47.21 | 1 & 447 | 2.1E^-11^ |
|  |  | QMCI | 0.10 | 31.24 | 1 & 291 | 5.2E^-8^ |
|  |  | ASPM | 0.14 | 62.11 | 1 & 386 | 3.3E^-14^ |
| Modelled | DIN | MCI | 0.21 | 461.3 | 1 & 1727 | 2.2E^-16^ |
|  |  | QMCI | 0.19 | 346 | 1 & 1448 | 2.2E^-16^ |
|  |  | ASPM | 0.22 | 485.9 | 1 & 1727 | 2.7E^-14^ |
|  | DRP | MCI | 0.12 | 231 | 1 & 1727 | 2.2E^-16^ |
|  |  | QMCI | 0.19 | 328.6 | 1 & 1448 | 2.2E^-16^ |
|  |  | ASPM | 0.14 | 292 | 1 & 1727 | 2.2E^-16^ |
